# Increased Missense Mutation Burden of Fatty Acid Metabolism Related Genes in Nunavik Inuit Population

**DOI:** 10.1371/journal.pone.0128255

**Published:** 2015-05-26

**Authors:** Sirui Zhou, Lan Xiong, Pingxing Xie, Amirthagowri Ambalavanan, Cynthia V. Bourassa, Alexandre Dionne-Laporte, Dan Spiegelman, Maude Turcotte Gauthier, Edouard Henrion, Ousmane Diallo, Patrick A. Dion, Guy A. Rouleau

**Affiliations:** 1 Montreal Neurological Institute and Hospital, McGill University, Montréal (Que), Canada; 2 Département de médecine, Faculté de médecine, Université de Montréal, Montréal (Que), Canada; 3 Département de psychiatrie, Faculté de médecine, Université de Montréal, Montréal (Que), Canada; 4 Centre de recherche, Institut universitaire en santé mentale de Montréal (Que), Canada; 5 Department of Human Genetics, McGill University, Montréal (Que), Canada; 6 Department of Neurology and Neurosurgery, McGill University, Montréal (Que), Canada; National Cancer Institute, National Institutes of Health, UNITED STATES

## Abstract

**Background:**

Nunavik Inuit (northern Quebec, Canada) reside along the arctic coastline where for generations their daily energy intake has mainly been derived from animal fat. Given this particular diet it has been hypothesized that natural selection would lead to population specific allele frequency differences and unique variants in genes related to fatty acid metabolism. A group of genes, namely *CPT1A*, *CPT1B*, *CPT1C*, *CPT2*, *CRAT *and *CROT*, encode for three carnitine acyltransferases that are important for the oxidation of fatty acids, a critical step in their metabolism.

**Methods:**

Exome sequencing and SNP array genotyping were used to examine the genetic variations in the six genes encoding for the carnitine acyltransferases in 113 Nunavik Inuit individuals.

**Results:**

Altogether ten missense variants were found in genes *CPT1A*, *CPT1B*, *CPT1C*, *CPT2* and *CRAT*, including three novel variants and one Inuit specific variant *CPT1A* p.P479L (rs80356779). The latter has the highest frequency (0.955) compared to other Inuit populations. We found that by comparison to Asians or Europeans, the Nunavik Inuit have an increased mutation burden in *CPT1A*, *CPT2* and *CRAT*; there is also a high level of population differentiation based on carnitine acyltransferase gene variations between Nunavik Inuit and Asians.

**Conclusion:**

The increased number and frequency of deleterious variants in these fatty acid metabolism genes in Nunavik Inuit may be the result of genetic adaptation to their diet and/or the extremely cold climate. In addition, the identification of these variants may help to understand some of the specific health risks of Nunavik Inuit.

## Introduction

Modern Inuit are descendants of the Dorset peoples from the second wave of migrations which took place over 1000 years ago [1]. Previous studies have established that these Inuit are genetically closest to the contemporary north-east Siberian populations [2], and belong to one of the three native ancestral groups that populated the Americas. The ancestors of today’s Nunavik (a region of northern Quebec) Inuit had migrated from Alaska to the east across the far north. With a population of barely 10,000 individuals, Nunavik Inuit form a very isolated population whose ancestral genetic profiles are likely to be well preserved until today. The Inuit enrolled in this study were recruited from 13 of the 14 inhabited villages, and they represent over 1% of the Nunavik Inuit population. As a part of their unique lifestyle, Nunavik Inuit derive approximately 75% of their daily energy intake from animal fat [3]. The traditional diet (high in fat and low in carbohydrates) of Nunavik Inuit suggests that they have high rates of gluconeogenesis, which is supported by their larger liver size compared to other populations [3].

The oxidative processing of fatty acids is critical for generating energy from a diet enriched with animal fat. Several enzymes are involved in the metabolism of fatty acids, including the carnitine acyltransferases [4]. The carnitine acyltransferase gene family is comprised of six members which encode for three types of enzymes: (1) carnitine palmitoyltransferases (CPTs) encoded by *CPT1A*, *CPT1B*, *CPT1C* and *CPT2*; (2) carnitine acetyltransferase (CrAT) encoded by *CRAT*; and (3) carnitine octanoyltransferase (CrOT) encoded by *CROT* [4]. The genomic features of these six genes are listed in **Table 1.** CPTs include two members, CPT-I and CPT-II. As a rate-limiting step in fatty acid oxidation, CPT-I converts long-chain fatty acyl molecules into their corresponding acylcarnitines, which are then transported across the inner mitochondrial membrane for beta-oxidation. CPT-II is responsible for the reversal of this process [5]. CrOT and CrAT catalyze the reversible transfer of fatty acyl groups between CoA and carnitine, and therefore determine the acetyl-CoA/CoA ratio [6].

**Table 1:** Genomic features of carnitine acyltransferase genes.

	Encode	Genomic region of longest isoform (number of isoforms)	Number of exons	HGMD mutation numbers from of all isoforms	Loss of function variant* from EVS
***CPT1A***	carnitine palmitoyltransferase-I (CPT-I) liver-type	chr11:68522088–68609399 (2)	20	29 (missense); 1 (splicing); 5 (indel)	3
***CPT1B***	CPT-I muscle-type	chr22:51007290–51017096 (4)	21	2 (missense); 1 (splicing)	9
***CPT1C***	CPT-I brain-type	chr19:50194365–50216988 (2)	20	N/A	4
***CPT2***	carnitine palmitoyltransferase—II (CPT-II)	chr1:53662101–53679869 (1)	5	65 (missense); 4 (splicing); 20 (indel)	3
***CRAT***	carnitine acetyltransferase (CrAT)	chr9:131857073–131873070 (1)	15	N/A	3
***CROT***	carnitine octanoyltransferase (CrOT)	chr7:86974951–86989425 (3)	18	N/A	12

HGMD: Human Gene Mutation Database, including all published gene mutations responsible to human inherited disease.

EVS: NHLBI Exome sequencing project exome variant server, including approximately 4,294 European Americans and 2,200 African Americans.

*Including nonsense mutations, frameshift mutations, splicing donor and acceptor site mutations.

CPT-I and II deficiencies are genetic disorders that are characterized by the reduced CPT enzyme activity which can lead to severe consequences, such as liver failure and renal problems [5, 7, 8]. Although these deficiencies are commonly caused by autosomal recessive mutations in *CPT1A* and *CPT2*, heterozygous carriers of *CPTs* mutations can also be symptomatic [9, 10].

Considering their critical roles in lipid metabolism and energy generation, the six genes encoding carnitine acyltransferases may be subjected to evolutionary pressure, especially in populations such as the Inuit, which require an increased enzyme activity because of their special diet and extreme living conditions. Recent studies have shown that the diet of Nunavik Inuit led to changes in specific biomarkers. For instance, Nunavik Inuit have very high levels of n-3 polyunsaturated fatty acids (PUFA), especially in the elderly [11], as well as high levels of plasma LDL cholesterol [12]. It is possible that these changes may be partially due to the variations in the carnitine acyltransferase genes, which can affect their enzymatic activity. Because of these biochemical characteristics, it is important to study the genes that are critical to fatty acid metabolism. In addition to providing insight about the Nunavik Inuit adaptation, the identification of variants in these genes may also help to understand some of their specific health risks. To examine this hypothesis, we used exome sequencing and SNP array genotyping to investigate genetic variants across these six genes in a cohort of Inuit from Nunavik.

## Materials and Methods

### Subjects

We recruited 113 Inuit individuals (62 males and 51 females, mean age 52 years) from 13 inhabited villages of Nunavik. The study was approved by Comité d'éthique de la recherche du Centre hospitalier de l'Université de Montréal (ND 04.101) (Québec, Canada) and the Nunavik Nutrition and Health Committee (Québec, Canada). Individual written consent was obtained from each participant before entering this study.

### Exome sequencing and coding variant identification from six carnitine acyltransferase genes

The genomic DNA of 113 Nunavik Inuit individuals was extracted from peripheral blood lymphocytes using Gentra Systems PUREGENE DNA purification kit (Qiagen). A 50 μl DNA sample at a concentration of 100 ng/μl from each sample was captured by Agilent SureSelect 50mb/V4 capture kit. The library was subsequently sequenced at 100 bp pair-end using Illumina Hiseq 2000, with 3 samples per lane to ensure an average coverage depth of 100-fold.

Raw fastq files were aligned to NCBI human reference GRCh37 using Burrows-Wheeler Aligner (BWA) [13], with all PCR duplicates removed from the alignments. The aligned reads were converted to binary format for further analysis using Sequence Alignment/Map (SAM) tools [14]. Single nucleotide variant (SNV) and insertion/deletion (indel) calling were performed using Genome Analysis Toolkit (GATK) version 2.7 [15]. Variant annotation was performed using ANNOVAR program [16] with references to GRCh37/hg19, dbSNP version 132, 1000 Genomes project (1KGP) (2012 data release) [17], 69 Complete Genomics (2012 update) and exome variant server (EVS) with approximately 6,500 exomes (NHLBI-ESP project, 2013 update) [18]. Finally, variant segregation analysis was performed using an in-house segregation program, using more than 1,000 exomes of different ethnicities from our lab as controls.

In the variant analysis, intronic, intergenic and non-coding variants were excluded because of insufficient coverage. Quality filters were set at sequencing depth ≥20x and variant frequency ≥25%, with genotype quality ≥10. Rare variants were defined as variants with minor allele frequency (MAF) ≤0.01 in the aforementioned public databases. All potential missense and splicing site variants from the genes *CPT1A*, *CPT1B*, *CPT1C*, *CPT2*, *CRAT* and *CROT* were examined, and were further validated by manual inspection using the Integrative Genomics Viewer 1.4 (IGV) [19] and Sanger sequencing.

### SNP genotyping and common variant identification from six carnitine acyltransferase genes

SNP genotyping was performed on 113 Nunavik Inuit, using Illumina HumanOmniExpress-12 SNP array, which contains 730,525 SNPs. Raw data was processed and analyzed using Illumina GenomeStudio and quality control was performed by PLINK 1.07 [20]. SNPs within each gene region and 2,000 bp up/downstream flanking regions were extracted from all individuals. Variant concordance within these regions was calculated between data from exome sequencing and SNP array genotyping. Allele frequencies and Hardy–Weinberg equilibrium (HWE) of all selected SNP were calculated using PLINK. Calculation of linkage disequilibrium (LD) and haplotype analysis were performed using Haploview 4.2 [21].

### Statistical analysis

Multidimensional scaling (MDS) and Admixture analyses [22] were performed on the Nunavik Inuit genotype data against HapMap genotype data from Asian (CHB and JPT), European (CEU) and African (YRI) populations to assess the mixture of Nunavik Inuit population.

Missense variations of the carnitine acyltransferase genes were retrieved from 286 Chinese (CHB) and Japanese (JPT) from the 1KGP database and European descendants from EVS, to serve as controls.

Mutation burden is defined as the average number of rare (MAF ≤0.01) missense variant alleles per person. To examine whether there is an increased mutation burden in the selected genes in Nunavik Inuit compared to other populations, we performed a binomial test to compare rare allele frequencies between Nunavik Inuit and 1KGP Asians. The Adaptive Permutation test was also performed to compare the mutation burden in Nunavik Inuit with Asians. The Permutation test was performed five times based on the number of variant-containing genes, with Bonferroni correction for multiple comparisons applied and p <0.01 was set as statistically significant.

We also calculated two F-statistics (*F*_*ST*_ and *F*_*IS*_) scores based on variants identified in the carnitine acyltransferase genes. The *F*_*ST*_ value provides an indication about the population genetic difference that is due to the genetic drift between subpopulations. The *F*_*IS*_ value measures the proportion of total inbreeding within a population that is due to non-random mating within subpopulations [23]. To further compare the impact of these variants in Nunavik Inuit to other populations, we generated a scatterplot using the R package “ggplot2” of all functional variants identified in *CPT1A*, *CPT1B*, *CPT1C*, *CPT2* and *CRAT* in Nunavik Inuit, 1KGP Asians and EVS Europeans. Mann-Whitney U test was performed to compare the PolyPhen-2 scores of all rare missense variants found in Nunavik Inuit and 1KGP Asian populations, with p <0.05 as significant.

## Results

### Data quality control

**Fig 1:**
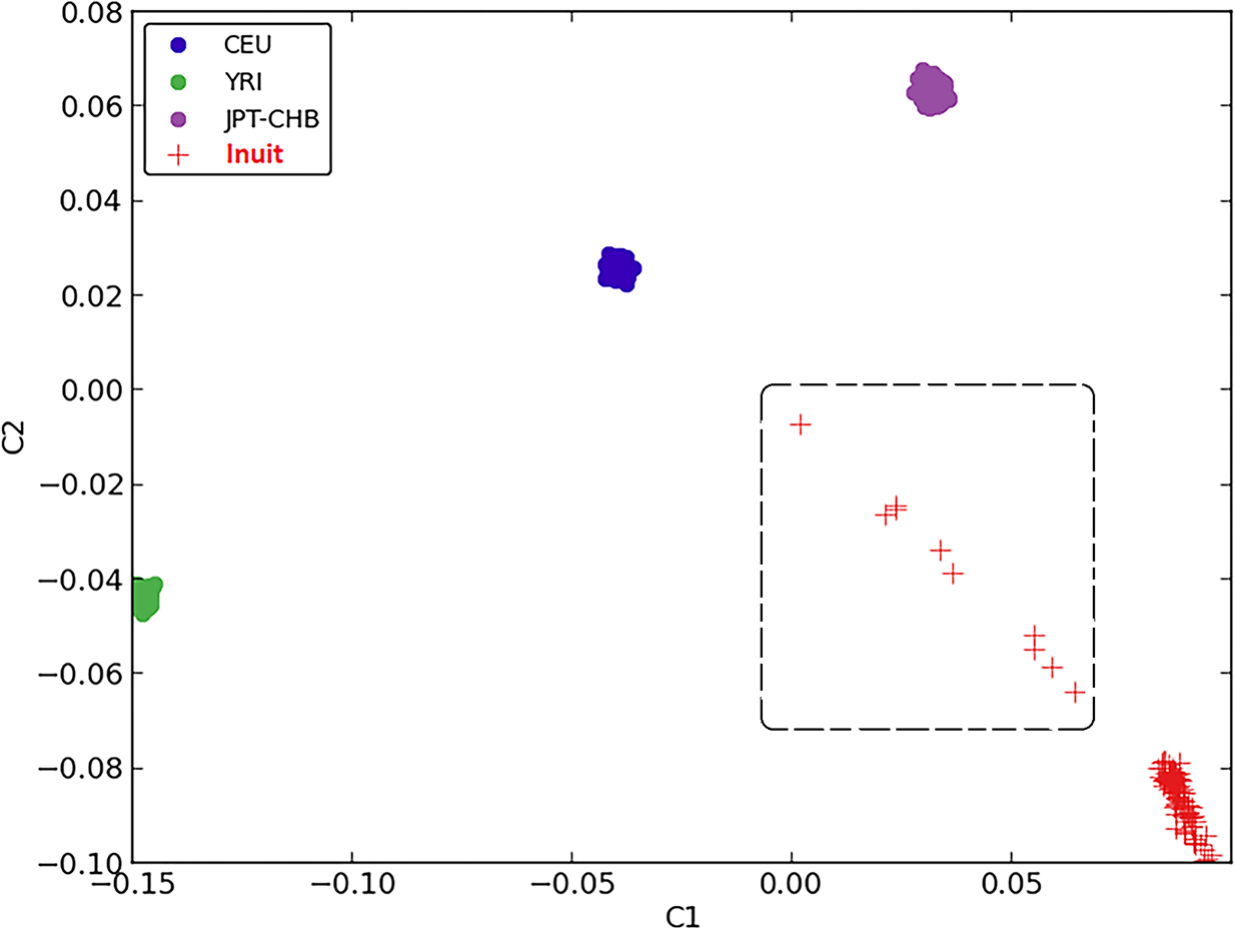
MDS plot showing the distinctive ethnicity relationships of CEU, YRI, JPT-CHB and Nunavik Inuit. Individuals in the dashed-line box have a mixture of both Inuit and European genomes.

Using the data from the HumanOmniExpress SNP array, four individuals with sex mismatch were identified by PLINK and removed from the subsequent analysis. After MDS testing, the genomes of nine additional individuals were found to have a mixture of both Inuit and European genomes and were therefore also removed from subsequent analysis. This mixture is probably the results of intermarriages between Nunavik Inuit and French-Canadians. Therefore a total of 100 Inuit samples were included in the genetic/statistical analysis (Fig 1).

Based on GATK's DepthOfCoverage Walker [15] program, the average coverage depth of the coding regions of *CPT1A*, *CPT1B*, *CPT1C*, *CPT2*, *CRAT* and *CROT* were 102X, 134X, 107X, 102X, 101X and 80X, respectively. The percentage of targeted region with coverage depth of more than 20X for each gene were 92.7%, 97.3%, 99.6%, 94.1%, 98.6% and 90.2%, respectively (S1 Fig).

Of the 6 carnitine acyltransferase genes, coding variants were identified only in *CPT1A*, *CPT1B*, *CPT1C*, *CPT2* and *CRAT* in Nunavik Inuit. Since no coding variants were found in *CROT*, all subsequent analyses and discussions were focused on the other 5 genes. Across these genes, 16 variants were identified by both the exome sequencing and SNP array, 4 of which were exonic variants. The concordance rate of the exonic variants between the two platforms was 100%, while for the intronic variants it was 77% (S1 Table). VCF (Variant Call Format) files of the exome sequencing data and PLINK genotype files of the SNP array covering the carnitine acyltransferase gene regions can be found in S1 Archive.

### Missense variants of the carnitine acyltransferase genes in different populations

In the 100 individuals included in the analysis, 10 missense and 3 synonymous variants were found in 5 carnitine acyltransferase genes. Three missense variants, *CPT1C* p.T265M, *CPT2* p.R477W, *CRAT* p.S78F and one synonymous variant *CPT1A* p.V616V, were unique to the Nunavik Inuit population (Table 2 and S2 Table). In addition, an Inuit specific variant *CPT1A* p.P479L was also observed. These five variants and a rare synonymous *CRAT* p.A575A variant were validated by Sanger sequencing (S2 Fig). The frequencies of the 10 missense variants were compared between the Nunavik Inuit, Asian, European and African populations. *In silico* prediction using PolyPhen-2 [24] and MutationTaster [25] suggested that the 4 Inuit specific missense variants are potentially deleterious (Table 3). Even with the exclusion of the Inuit high frequency variant *CPT1A* p.P479L (rs80356779), the Polyphen-2 scores of all the rare variants found in Nunavik Inuit and 1KGP Asian population were still significantly higher (Mann-Whitney U test, P = 0.02) for Nunavik Inuit (Table 4 and S3 Fig).

**Table 2:** Coding variants of carnitine acyltransferase genes discovered in Nunavik Inuit.

Gene	Variant	Variation type	Annotation
***CPT1A***	p.V616V	synonymous	novel
***CPT1A***	p.P479L	missense	rs80356779
***CPT1A***	p.F417F	synonymous	rs2228502
***CPT1B***	p.E531K	missense	rs470117
***CPT1B***	p.S427C	missense	rs8142477
***CPT1B***	p.I66V	missense	rs3213445
***CPT1C***	p.T265M	missense	novel
***CPT2***	p.F352C	missense	rs2229291
***CPT2***	p.V368I	missense	rs1799821
***CPT2***	p.R477W	missense	novel
***CRAT***	p.A603P	missense	rs17459086
***CRAT***	p.A575A	synonymous	rs375414636
***CRAT***	p.S78F	missense_splicing	novel

**Table 3:** Variant frequencies and deleterious score predictions of carnitine acyltransferase genes.

Gene	Protein coding variants	SNP	MutationTaster	PolyPhen v2	Frequency* in Nunavik Inuit (100)	Frequency in CHB-JPT (286)	Frequency in CEU (178)	Frequency in YRI (250)
***CPT1A***	p.P479L	rs80356779	0.997899	1	95.5%	0	0	0
***CPT1B***	p.E531K	rs470117	0.606166	0.303	30.5%	48.3%	46.5%	8.8%
***CPT1B***	p.S427C	rs8142477	0	0	67.5%	51.7%	93.3%	25.8%
***CPT1B***	p.I66V	rs3213445	0.251908	0	17.5%	35.8%	4.9%	10.2%
***CPT1C***	p.T265M		0.999717	0.998	0.5%	0	0	0
***CPT2***	p.F352C	rs2229291	0.999596	0.999	28.0%	20.0%	0	1.3%
***CPT2***	p.V368I	rs1799821	0.06145	0.001	52.5%	77.5%	54.9%	25.2%
***CPT2***	p.R477W		0.997459	1	3.0%	0	0	0
***CRAT***	p.A603P	rs17459086	0.993409	0.013	9.0%	7.0%	1.7%	0.8%
***CRAT***	p.S78F		0.999657	0.906	2.5%	0	0	0

CHB, JPT, CEU, YRI are from 1KGP.

*Based on the derived allele from the reference genome.

**Table 4:** Deleterious scores of all rare missense variants in carnitine acyltransferase genes found in Nunavik Inuit and 1KGP Asians.

	PolyPhen v2	Derived allele frequency (%)
**Nunavik Inuit (100)**		
***CPT1A*: p.P479L**	1	95.5
***CPT1C*: p.T265M**	0.998	0.5
***CPT2*: p.R477W**	1	3
***CRAT*: p.S78F**	0.906	2.5
**1KGP Asians (286)**		
***CPT1A*: p.I491T**	0	0.35
***CPT1B*: p.C659W**	1	0.35
***CPT1C*: p.R514Q**	1	0.35
***CPT1C*: p.Q97H**	0.08	0.35
***CPT2*: p.S122F**	0.018	0.6
***CRAT*: p.V411M**	0.803	0.35

### Mutation burden test and F-statistics of variants in the carnitine acyltransferase genes in Nunavik Inuit

In order to characterize the genetic profile of the carnitine acyltransferase genes in Nunavik Inuit, the mutation burden for each gene was calculated in the Inuit and compared to the mutation burden in 286 Asians (CHB and JPT) from 1KGP. Nunavik Inuit had significantly more mutated alleles per individual in the *CPT1A*, *CPT2* and *CRAT* genes than Asians (Binomial test, p <0.01). Permutation test showed similar results with marginally significant p values in *CPT2* and *CRAT* (not significant after Bonferroni correction) (Table 5).

**Table 5:** Mutation burden scores of identified rare mutations (MAF<0.01) of carnitine acyltransferase genes in Nunavik Inuit and 1KGP Asians.

Gene	Variants	# variant alleles/Inuit (100)	# variant alleles/Asian (286)	Inuit/Asian ration	Base-pair sequenced	P^1^	P^2^
***CPT1A***	p.P479L*, p.I491T	1.91	0.007	272.80	2319	**2.2e-16**	**0.000006**
***CPT1B***	p.C659W	0	0.007	0.00	2316	1	0.75
***CPT1C***	p.T265M*, p.Q97H, p.R514Q	0.01	0.014	0.71	2409	1	1
***CPT2***	p.R477W*, p.S122F	0.06	0.017	3.53	1974	**0.003**	0.03689
***CRAT***	p.S78F*, p.V411M	0.05	0.007	7.14	1815	**0.00076**	0.01305

^1^: Binomial test, Bonferroni corrected, significant p<0.01.

^2^: Empirical p-value of permutation test, 10^5^ permutations.

*Variants only identified in Inuit.

*F*_*ST*_ and *F*_*IS*_ scores were calculated for 100 Nunavik Inuit and 83 HapMap Asians (CHB and JPT). The *CPT1A* p. P479L variant had the highest *F*_*ST*_ value (0.92), and the mean *F*_*ST*_ of the 4 Inuit specific variants was also statistically significant (0.84, p<0.01). However, the mean *F*_*IS*_ of all variants did not significantly deviate from zero after 1,000 randomizations (-0.0092) (Table 6).

**Table 6:** The *F*_*ST*_ and *F*_*IS*_ value of 13 variants in the population containing Nunavik Inuit and HapMap Asians.

Variant	SNP	*F*_*ST*_	*F*_*IS*_
***CPT1A*: p.V616V**		0.0196	-0.04
***CPT1A*: p.P479L**	rs80356779	0.9171*	-0.0452
***CPT1A*: p.F417F**	rs2228502	0.0506	-0.1067
***CPT1B*: p.E531K**	rs470117	0.0414	-0.0273
***CPT1B*: p.S427C**	rs8142477	0.034	0.0382
***CPT1B*: p.I66V**	rs3213445	0.0453	0.0114
***CPT2*: p.F352C**	rs2229291	0.0298	-0.0925
***CPT2*: p.V368I**	rs1799821	0.0546	0.0349
***CPT2*: p.R477W**		0.0146	-0.0297
***CRAT*: p.A603P**	rs17459086	0.0045	-0.0227
***CRAT*: p.A575A**	rs375414636	0.0097	-0.0196
***CRAT*: p.S78F**		0.0122	-0.0246
***CPT1C*: p.T265M**		0.0024	-0.0048
**Mean (all variants)**		0.1833	-0.0092
**Mean (missense variants)**		0.1907	-0.0053
**Mean (Inuit specific variants)**		0.8401*	-0.0393

*F*_*ST*_: The fixation index, which is the proportion of total genetic variance contained in a subpopulation relative to the total genetic variance. The value of *F*_*ST*_ ranges from 0 to 1, the higher value implies higher degrees of population differentiation.

*F*_*IS*_: The inbreeding coefficient, which is the proportion of genetic variance in the subpopulation contained in an individual.

*Statistical significance, 1000 randomizations

**Fig 2:**
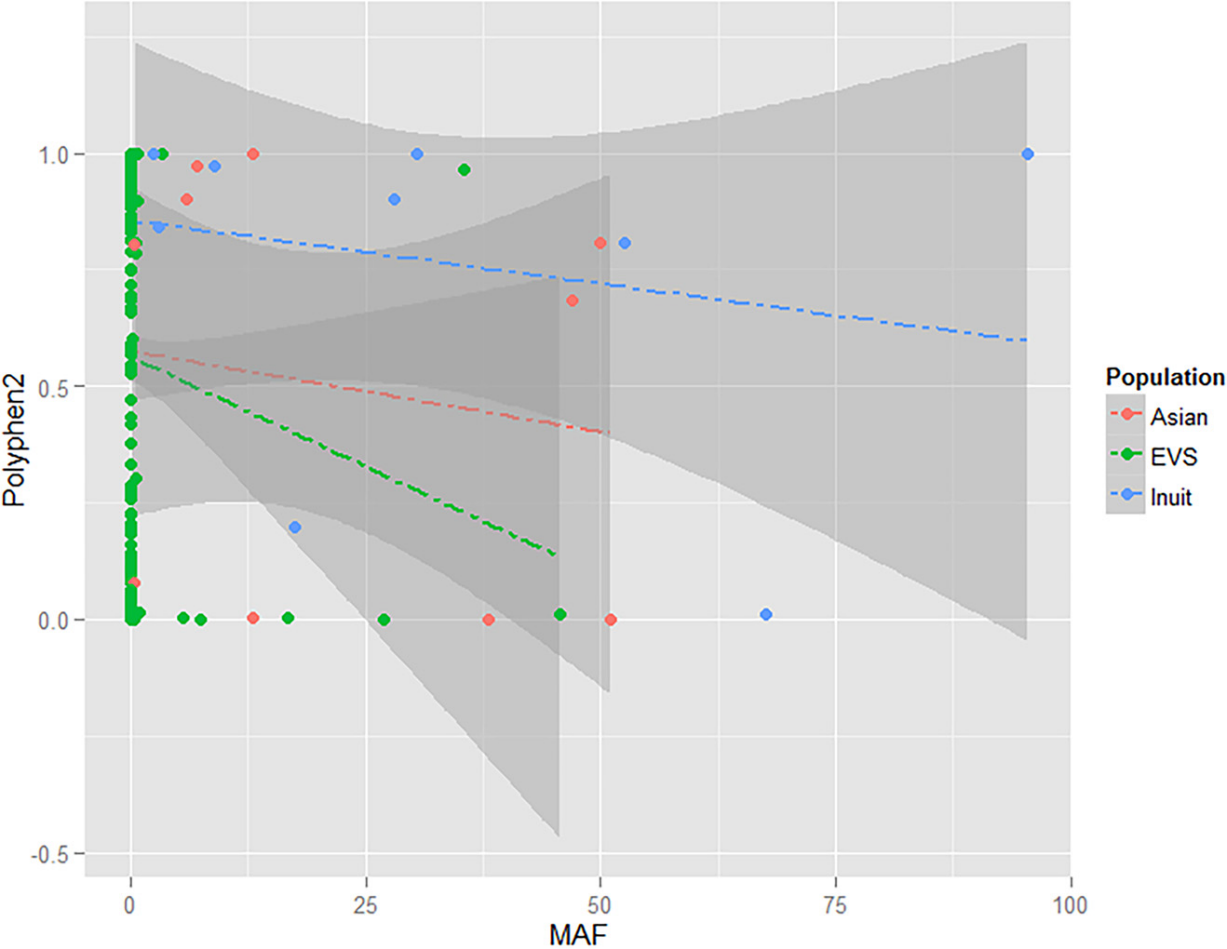
Scatterplot of the frequencies and deleterious scores of *CPT1A*, *CPT1B*, *CPT1C*, *CPT2* and *CRAT* missense variants. Variants were extracted from 286 Asians from 1KGP, EVS populations comprising 4,294 European descendants and 100 Nunavik Inuit.

Fig 2 depicted the frequencies and deleterious scores of the functional variants of the carnitine acyltransferase gene in different populations. The allele frequencies of all missense variants in European descendants (EVS database), Asians (1KGP) and the Nunavik Inuit were plotted against the PolyPhen-2 score of each variant. Variants with PolyPhen-2 score >0.8 (predicted to be highly deleterious) were presented at higher frequencies in the Nunavik Inuit compared to Asians and European descendants (Fig 2).

### Common missense variants in the carnitine acyltransferase genes

**Fig 3:**
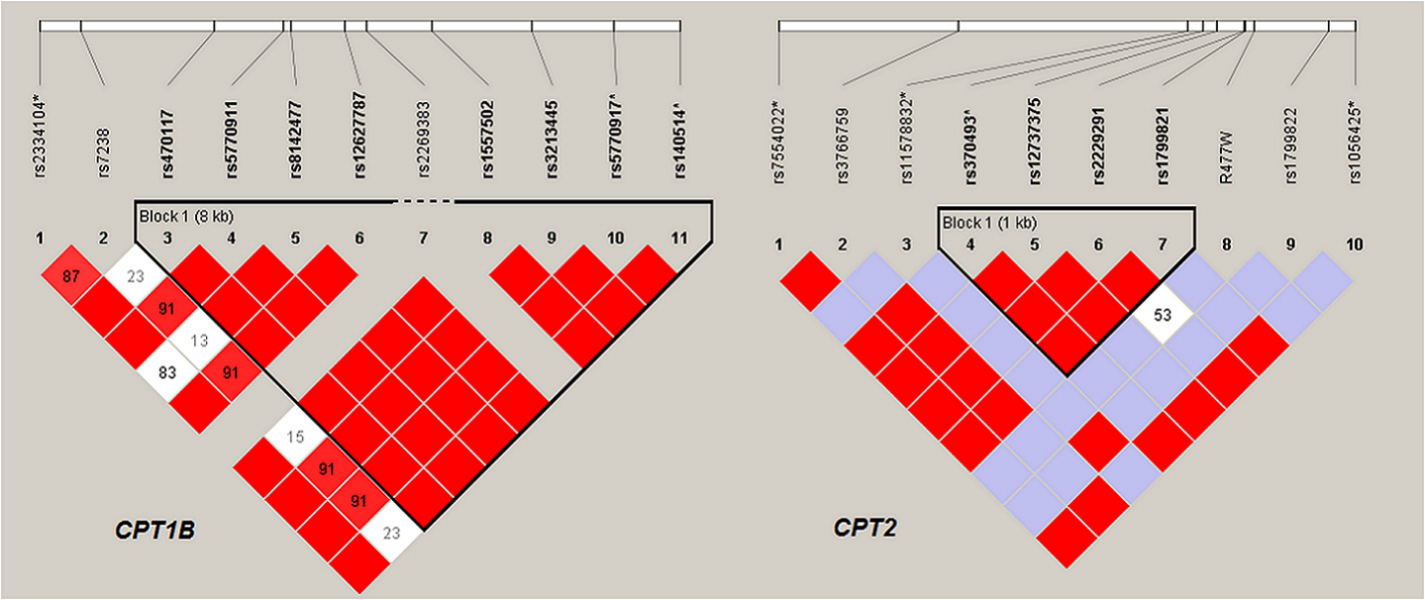
Pairwise linkage disequilibrium (LD) diagram for *CPT1B* and *CPT2* in Inuit. LD block was delineated using confidence intervals (Gabriel et al), and 0.6–0.98 for strong LD. Variants of MAF<0.05 were indicated with dashed-line. Variants were extracted from exome sequencing and SNP array data, with the asterisk indicated variants only genotyped by the SNP array. D value <1 was shown in the box, with red color indicated LOD ≥2.

HWE testing did not show a significant deviation for any of the exonic variants identified in the 5 genes. Haplotype analysis found that 3 common Nunavik Inuit *CPT1B* missense variants (rs470117, rs8142477 and rs3213445) were in an 8 kb LD block. The frequency of this haplotype in the Inuit (0.66) was significantly higher than in Asians (0.46) (Binomial test, p = 7.501e-05). In *CPT2*, two common missense variants rs2229291 (p.F352C) and rs1799821 (p.V368I) are in a 1 kb LD block (Fig 3). These variants along with the Nunavik Inuit specific variant p. R477W form four major haplotypes in the studied cohort: T-G-C (45%), T-A-C (24%), G-A-C (28%) and T-G-T (3%), respectively. While in Europeans and Africans, only the T-G-C and T-A-C haplotypes were found. In Nunavik Inuit the G-A-C haplotype frequency is 28% compared to 10% in Asians [26], and absent in Europeans (Binomial test, p = 3.481e-07).

## Discussion

### Carnitine acyltransferase gene variants in the Nunavik Inuit

Our results indicate that Nunavik Inuit are genetically distinct from European, African and Asian populations, with the closest relationship to Asians. In 100 Nunavik Inuit we found three novel missense variants and a known Inuit specific missense variant in 4 carnitine acyltransferase genes. The rare missense mutations are significantly more deleterious and their frequencies are significantly higher in Nunavik Inuit, compared to other populations. Interestingly, we didn’t find *CROT* missense variants in either Nunavik Inuit or Asians.

Mutation burden testing shows that mutations in the *CPT1A*, *CPT2* and *CRAT* genes are significantly more frequent in Nunavik Inuit compared to Asians. The mean *F*_*ST*_ value (0.18) of all variants indicates a great genetic differentiation between Nunavik Inuit and Asians (*F*_*ST*_ >0.15); it is also noteworthy that the 4 Inuit specific variants are indicating an even higher degree of differentiation for this population (*F*_*ST*_ >0.25) (Table 6). However the *F*_*IS*_ value shows no statistical significance, which may due to the limited number of variants available. Nevertheless, the negative mean *F*_*IS*_ value suggests that there could be an excess of heterozygotes within the populations.

### Nunavik Inuit population specific variants

#### *CRAT* and *CPT2*

The *CRAT* p.S78F mutation is located at the splicing site and is predicted to affect splicing, potentially to leading to a loss of function of CrAT and carnitine acetyltransferase deficiency. Moreover, in a muscle-specific CrAT knockout mouse model, CrAT acts as a modulator of whole-body glucose homeostasis and metabolic flexibility [27]. The Inuit appear to tolerate drastic changes in metabolic homeostasis, hence the loss of CrAT function may be beneficial to them. Symptomatic CPT-II deficiency is usually caused by homozygous or compound heterozygous mutations in *CPT2*. The *CPT2* p.R477W mutation found in the Nunavik Inuit is likely to lead to the loss of function as it is located in a highly conserved region where mutations known to cause CPT-II deficiency occur [28, 29]. It is possible that heterozygote loss of function mutations in *CPT2* may have a beneficial effect on the enzyme activity in Nunavik Inuit.

#### The high frequency *CPT1A* p.P479L variant

In general, missense mutations are rare in *CPT1A*; yet in some Inuit populations, the p.P479L loss of function mutation has a high frequency, ranging from 44% to 83% from Alaska to Greenland [30, 31]. In our study of the Nunavik Inuit, the p.P479L mutation has an allele frequency of 95.5%, the highest reported to date. The frequency of this variant seems to increase from the west to the east along the arctic tree line and from inland to the shore (S4 Fig), correlating with the migration timeline and with the higher consumption of animal fat of residents near the shore. The *CPT1A* p.P479L mutation is significantly more frequent among Nunavik Inuit compared to 243 Kivalliq Inuit (western Nunavut) (χ2 = 15.629, p = 0.0004) [31]. Interestingly, the *CPT1A* p.P479L mutation is absent from all other worldwide populations, including East Asians (Table 3). This variant was initially believed to cause CPT-I deficiency, since the first discovery was made in a Canadian First Nation man with myopathy [32], a typical subtype of CPT1A deficiency. Therefore, this variant has been included in the Alaska newborn screening protocol [33]. In addition, in Canadian Inuit and First Nation families with severe CPT-I deficiency, the only missense mutation found in the *CPT1A* gene was the homozygous p.P479L variant [34]. It was further demonstrated that the presence of the p.P479L variant in both *CPT1A* alleles resulted in reduced CPT-I activity in cultured fibroblasts and affected malonyl-CoA interaction with CPT1A [10]. However, other studies of this variant in Yup'ik Eskimos and Greenland Inuit yielded different results. In these studies, the L479 allele was reported to be associated with infant mortality [35], with impaired fasting tolerance [36], reduced adiposity [37] and with higher levels of HDL-cholesterol and apoA-I cardioprotective factors [38]. Nevertheless, it is important to note that Yup'ik Eskimos and Canadian Inuit are genetically different [39]. The reduced CPT1A activity associated with the homozygous state of p.P479L variant in Inuit may indicate two scenarios: A) lower activity of the p.P479L variant is beneficial for a state of permanent ketoadaptation in Inuit [34] or B) another Inuit specific variant in the regulatory region may serve as a rescue factor to the enzyme activity.

Although different disorders and traits associated with this variant were reported in different studies, the presence of this variant in very high frequency in Nunavik Inuit suggests that it is likely to be beneficial in this population. For example, it may help the arctic residents to adapt to the extremely cold environment and/or their ketogenic diet. This assumption is supported by the mean age difference found in previous studies and in the current study. Previously, it was reported that the p.P479L variant frequency is higher among Inuit newborns and children [31, 35]. However, the current study shows that among older populations (mean age of 52 years at enrollment) the L479 allele remains predominant. Furthermore, infant mortality rates are higher in Nunavik than in Nunavut (25 vs 14.6 per 1,000 live births) and Quebec (5 per 1,000 live births), but lower in Kivalliq (32.3 per 1,000 live births) [40]. These conflicting data suggest that further investigation is needed to determine whether there is an association between the p.P479L variant and infant death. Since the p.P479L variant is absent from Chinese and Japanese populations, it probably occurred more recently, after the migration across the Bering Strait of their Asian ancestors. Genotyping the p.P479L variant in Siberian and Mongolian populations will be interesting to trace the origin and the occurrence of this variant.

### *CPT1B* and *CPT2* haplotypes in the Nunavik Inuit population

The mutation burden and tolerance tests did not show any excess of rare missense *CPT1B* variant in Nunavik Inuit. Nevertheless, the relative frequencies of different *CPT1B* locus rs470117/rs8142477/rs3213445 containing haplotypes found in the Nunavik Inuit are different from those seen in Asian populations. Interestingly, this haplotype is in the same LD block as the SNP rs5770917, which was reported to be associated with narcolepsy in the Japanese population [41, 42]. Given that the Inuit live in the far north with the midnight sun and the polar night, variants in this locus may therefore have some roles in sleep, possibly benefiting the Inuit while predisposing to narcolepsy in Japan.

*CPT2* missense mutations are rare in the general population, suggesting that the variations in the *CPT2* gene seen in Nunavik Inuit may be functional, possibly related to the energy metabolism requirements unique to this population. The haplotype containing p.F352C and p.V386I variants was previously named as a thermolabile CPT-II variant [43] with decreased CPT-II activity. It was further reported as a risk factor for infection-induced acute encephalopathy and for continuous high-grade childhood fever, which leads to a systemic and metabolic energy crisis in Japanese and Chinese populations [26, 43, 44]. The *CPT2* p.F352C variant was also reported in three individuals from one Inuit family with CPT-II deficiency [34]. However, there was no clinical description of acute encephalopathy in this family. Since this variant is temperature sensitive, it may act differently or even lead to higher enzyme activities in cold temperatures, which may explain their increase of the thermolabile haplotype frequency in the Inuit. Of note, encephalopathy was not reported in the Nunavik Inuit cohort, suggesting that the same genetic variation may be associated with different phenotypes in different populations.

In this study, we observed an increased frequency of rare divergent variants in the carnitine acyltransferases family of genes in Nunavik Inuit in Quebec, as compared to other populations. There are few missense mutations in these genes, suggesting that they do not tolerate variation. As Asians are thought to have common ancestors with the Inuit [45], the excess of variants in these genes in Nunavik Inuit also suggests an effect of selective pressure. It is possible that these variants are related to their high fat diet, as the carnitine acyltransferase genes are essential for fat metabolism.

Since data on metabolic measurements was not available in our cohort, we could not determine whether the high frequency of variations in the carnitine acyltransferase genes in Nunavik Inuit affects their enzyme levels and activities. Our cohort was comprised of healthy Nunavik Inuit individuals and individuals with a family history of brain aneurysms; none of them showed any of the severe symptoms caused by CPTs or CrAT deficiencies. We hypothesize that these variants, while being part of the Inuit ‘healthy genomes’, could be harmful in other populations. Further studies in other Inuit populations, with the inclusion of measurements of these enzymes’ levels and activity are necessary to confirm our results and conclusions.

## Supporting Information

S1 Archive Data of carnitine acyltransferase genes from exome sequencing and SNP array. (7Z)

S1 Fig The average coverage of gene *CPT1A*, *CPT1B*, *CPT1C*, *CPT2*, *CRAT* and *CROT* from the exome sequencing data of 113 Nunavik Inuit. (TIF)

S2 Fig Chromatograms of Sanger sequencing validation of 4 Inuit specific missense variants and 2 rare synonymous variants identified in Nunavik Inuit. *CPT1A* p.P479L variant was only shown in a selected of samples.
(TIF)

S3 Fig Rare missense variants from Nunavik Inuit and 1KGP Asians with their gradation profile of Polyphen-2 scores. (TIF)

S4 Fig The *CPT1A* p.P479L variant frequencies in different Inuit settlements in Canada and the migration patterns of Inuit. Original map from “*Canada's Relationship with Inuit*: *A History of Policy and Program Development”*.
(TIF)

S1 Table Variant concordance of *CPT1A*, *CPT1B*, *CPT1C*, *CPT2* and *CRAT* from SNP array and exome sequencing. ^1^Variant only in individuals with mixed ethnicity. ^2^Variant with MAF = 1 in Inuit, Asians and Europeans.
(DOCX)

S2 Table All exonic variants of *CPT1A*, *CPT1B*, *CPT1C*, *CPT2* and *CART* in 100 Nunavik Inuit. (DOCX)
