# Supplementary figures and images for: Increased Missense Mutation Burden of Fatty Acid Metabolism Related Genes in Nunavik Inuit Population
Source: PLoS One. 2015 May 26;10(5):e0128255. doi: 10.1371/journal.pone.0128255 (PMC4444093; doi:10.1371/journal.pone.0128255)

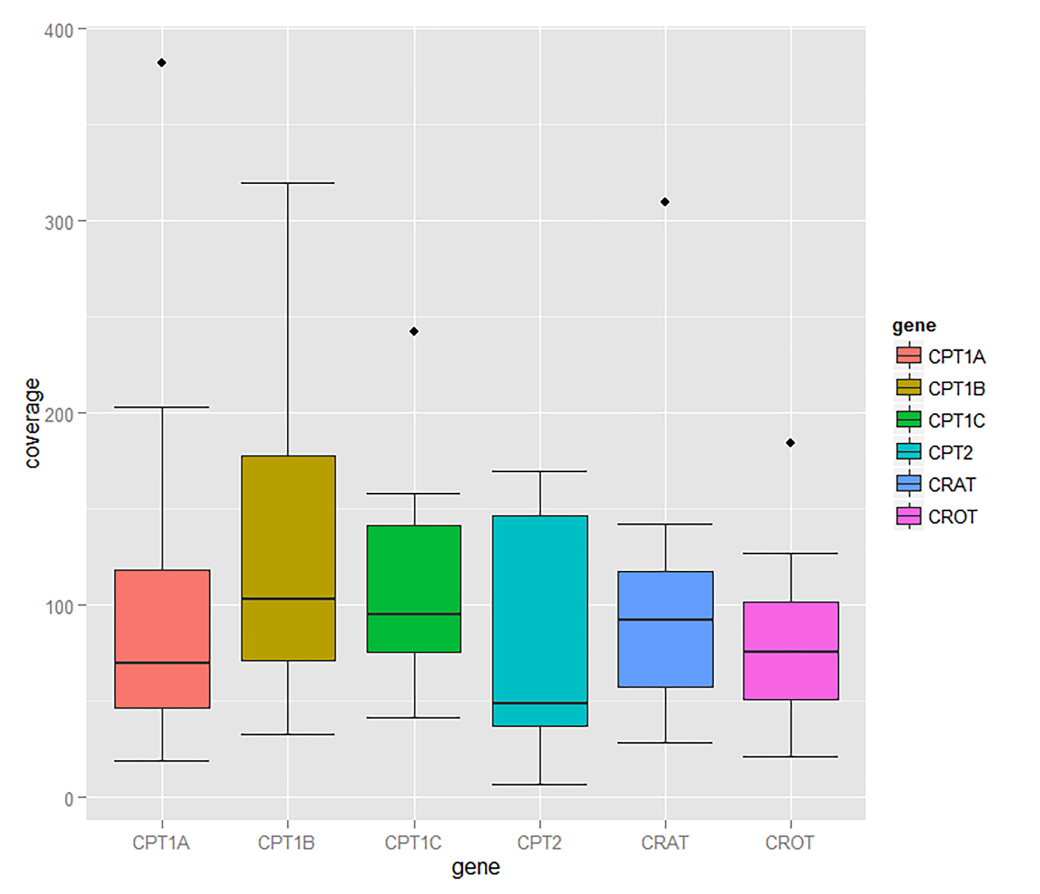

Supplement: S1 Fig — (TIF) [file pone.0128255.s002.tif]

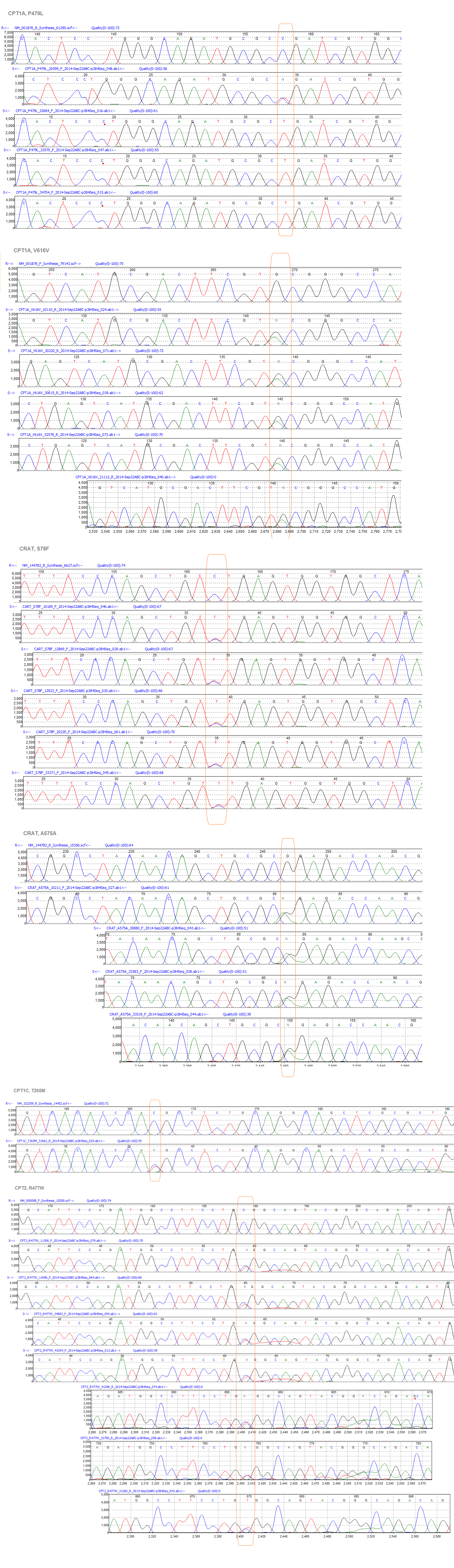

Supplement: S2 Fig — CPT1A p.P479L variant was only shown in a selected of samples. (TIF) [file pone.0128255.s003.tif]

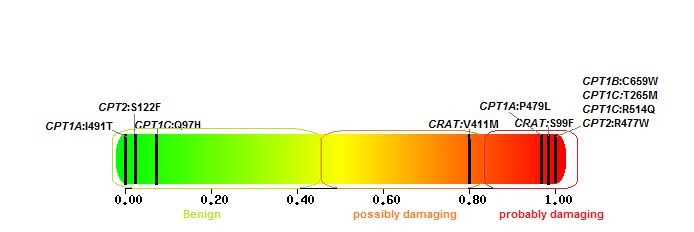

Supplement: S3 Fig — (TIF) [file pone.0128255.s004.tif]

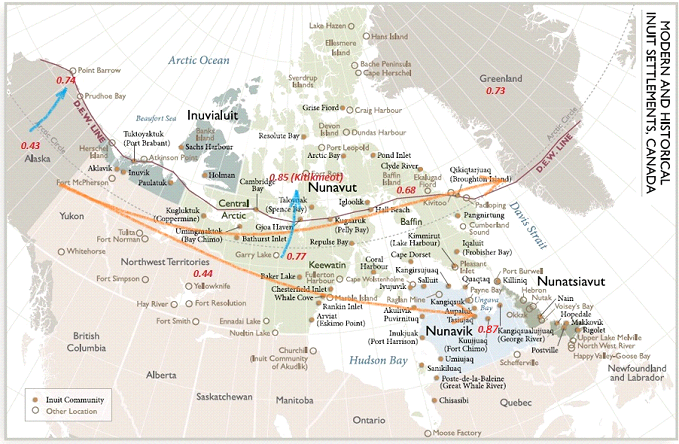

Supplement: S4 Fig — Original map from “Canada's Relationship with Inuit: A History of Policy and Program Development”. (TIF) [file pone.0128255.s005.tif]
