# Supplementary material for: Increased Missense Mutation Burden of Fatty Acid Metabolism Related Genes in Nunavik Inuit Population
Source: PLoS One. 2015 May 26;10(5):e0128255. doi: 10.1371/journal.pone.0128255 (PMC4444093; doi:10.1371/journal.pone.0128255)
Supplement: S1 Table — 1Variant only in individuals with mixed ethnicity. 2Variant with MAF = 1 in Inuit, Asians and Europeans. (DOCX) [file pone.0128255.s006.docx]

| **Variant** | **location** | **Gene** | **Success rate of genotyping (%)** | **Success rate of exome sequencing (%)** | **Variant concordance (%)** |
| --- | --- | --- | --- | --- | --- |
| **rs3019598** | Intronic | *CPT1A* | 100 | 100 | 100 |
| **rs7112615** | Intronic | *CPT1A* | 100 | 100 | 100 |
| **rs2305508** | Intronic | *CPT1A* | 100 | 99 | 91 |
| **rs2924674** | Intronic | *CPT1A* | 100 | 93 | 100 |
| **rs7238** | UTR-3 | *CPT1B* | 100 | 82 | 79 |
| **rs5770911** | Intronic | *CPT1B* | 100 | 100 | 100 |
| **rs12627787** | Intronic | *CPT1B* | 99 | 98 | 100 |
| **rs1557502** | Intronic | *CPT1B* | 100 | 43 | 65 |
| **rs3213445** | Exonic | *CPT1B* | 100 | 100 | 100 |
| **rs10407097** | Intronic | *CPT1C* | 100 | 35 | 80 |
| **rs3766759** | Intronic | *CPT2* | 100 | 15 | 40 |
| **rs12737375** | Intronic | *CPT2* | 100 | 80 | 86 |
| **rs1799821** | Exonic | *CPT2* | 100 | 100 | 100 |
| **rs1799822^1^** | Exonic | *CPT2* | 100 | 100 | 100 |
| **rs3118635^2^** | Exonic | *CRAT* | 100 | 100 | 100 |
| **rs10988209** | Intronic | *CRAT* | 100 | 45 | 73 |
|  | | | **Mean** | **Mean** | **Mean** |
| **Exonic variant** | | | 100 | 100 | 100 |
| **Intronic variant** | | | 99.9 | 73.3 | 77 |
