# Supplementary material for: Increased Missense Mutation Burden of Fatty Acid Metabolism Related Genes in Nunavik Inuit Population
Source: PLoS One. 2015 May 26;10(5):e0128255. doi: 10.1371/journal.pone.0128255 (PMC4444093; doi:10.1371/journal.pone.0128255)
Supplement: S2 Table — (DOCX) [file pone.0128255.s007.docx]

| **gene** | **variant position (hg19)** | **variant function** | **nucleotide change** | **amino acid change** | **SNP** | **1KGP frequency** | **Nunavik Inuit frequency (based on 200 alleles)** | **Genotyped** |
| --- | --- | --- | --- | --- | --- | --- | --- | --- |
| ***CPT1A*** | chr11:68530122 | Silent | c.G1848A | p.V616V | GCGACTTCGT[G/A]CGGGCCATGG | 0 | 0.04 |  |
| ***CPT1A*** | chr11:68548130 | Missense | c.C1436T | p.P479L | rs80356779 | 0 | 0.955 |  |
| ***CPT1A*** | chr11:68549340 | Silent | c.T1251C | p.F417F | rs2228502 | 0.91 | 1 |  |
| ***CPT1B*** | chr22:51009953 | Missense | c.G1591A (NM_152246) | p.E531K | rs470117 | 0.38 | 0.32 |  |
| ***CPT1B*** | chr22:51011376 | Missense | c.C1280G (NM_152246) | p.S427C | rs8142477 | 0.36 | 0.66 |  |
| ***CPT1B*** | chr22:51015838 | Missense | c.A196G (NM_152246) | p.I66V | rs3213445 | 0.17 | 0.185 | yes |
| ***CPT1C*** | chr19:50208286 | Missense | c.C794T | p.T265M | GTCACACCCA[C/T]GCCTCTGCAG | 0 | 0.005 |  |
| ***CPT2*** | chr1:53676401 | Missense | c.T1055G | p.F352C | rs2229291 | 0.06 | 0.275 |  |
| ***CPT2*** | chr1:53676448 | Missense | c.G1102A | p.V368I | rs1799821 | 0.5 | 0.535 | yes |
| ***CPT2*** | chr1:53676775 | Missense | c.C1429T | p.R477W | GGCCTTCCTG[C/T]GGCAGTACGG | 0 | 0.03 |  |
| ***CRAT*** | chr9:131857687 | Missense | c.G1807C (NM_000755) | p.A603P | rs17459086 | 0.04 | 0.1 |  |
| ***CRAT*** | chr9:131857769 | Silent | c.G1725A (NM_000755) | p.A575A | rs375414636 | 0 | 0.02 |  |
| ***CRAT*** | chr9:131866581 | Splicing | c.C233T (NM_000755) | p.S78F | TCCCAGCTGT[C/T]TGAGTGGTGG | 0 | 0.02 |  |
